# Efficacy of single high-molecular-weight versus triple low-molecular-weight hyaluronic acid intra-articular injection among knee osteoarthritis patients

**DOI:** 10.1186/s12891-020-03577-8

**Published:** 2020-08-15

**Authors:** Mohammad Hasan Bahrami, Seyed Ahmad Raeissadat, Mohsen Cheraghi, Shahram Rahimi-Dehgolan, Adel Ebrahimpour

**Affiliations:** 1 Physical Medicine and Rehabilitation Department and Research Center, Shohada-e-Tajrish Hospital, Shahid Beheshti University of Medical Sciences, School of Medicine, Tehran, Iran; 2 Clinical Development Research Center of Shahid Modarres Hospital, Physical Medicine and Rehabilitation Department and Research Center, Shahid Beheshti University of Medical Sciences, School of Medicine, Tehran, Iran; 3 Physical Medicine and Rehabilitation Department, Tehran University of Medical Sciences (TUMS), School of Medicine, Tehran, Iran; 4 Orthopedic surgery Department, Taleghani Hospital, Shahid Beheshti University of Medical Sciences, School of Medicine, No. 1998734383, Velenjak, Tehran, Iran

**Keywords:** Intra-articular injections, Cross-linked, Hyaluronic acid, Knee osteoarthritis

## Abstract

**Background:**

To compare intra-articular (IA) knee injections of a cross-linked high-molecular-weight hyaluronic acid (HMW-HA) with a linear low-molecular weight HA (LMW-HA) in terms of pain and functional improvement among knee osteoarthritis (OA) patients.

**Methods:**

In this single-blinded RCT, the patients were randomly divided into two groups for HA injections. The first group received an HMW-HA (Arthromac) injection, while the other received three weekly LMW-HA (Hyalgan) injections. Pain and function were assessed using the outcome measures including WOMAC, Lequesne and VAS indices, once prior to injection, as well as 2 and 6 months after injections.

**Results:**

A total of 90 patients were included. There was no significant difference in baseline characteristics including age and sex between the two groups. Our analysis showed that total WOMAC, Lequesne and VAS mean scores remarkably improved at both follow-up time-points compared to the baseline measurements (*p* < 0.001). There was no significant superiority between the two therapeutic protocols according to our outcome measures at any time-point of follow-up. The only except was about the improvement in WOMAC stiffness subscale that was significantly higher in LMW-HA group compared to HMW-HA (*p* = 0.021). Moreover, no significant difference was observed in minor complications and injection-induced pain scores between the two groups.

**Conclusion:**

This study proved that a single HMW-HA injection is as effective as multiple injections of LMW-HA counterparts in periods of 2 and 6 months follow-up.

This study protocol was registered in Iranian database of RCTs (IRCT; www.irct.ir) with the trial registration number IRCT20130523013442N24 and registration date 2018-07-13.

## Background

Osteoarthritis (OA) has been known to be the most common articular disease [1]. The prevalence of knee OA has doubled since the mid-twentieth century [2]. By examining the DALY among selected conditions throughout the world, knee and hip OA was determined to be at the 11th rank of global disability [3].

Treatments of knee are not considered to be a disease-modifying therapy [4]. medications including nonsteroidal anti-inflammatory drugs (NSAIDs), acetaminophen, duloxetine, opioids, topical NSAIDs and capsaicin are effective in reducing symptoms [5]. Intra-articular injection, can be carried out using corticosteroid [6], hyaluronic acid (HA) [7], ozone [8], plasma rich in growth factor (PRGF) [9], and Platelet-rich plasma (PRP) [10]. Physical agent modalities have also been investigated regarding knee OA [11, 12]. Many of international scientific associations have recommended Intra-articular hyaluronic acid (IA-HA) injections as part of knee OA treatment [13]. .HA has been compared with ozone [14], PRP [15], PRGF [16], and corticosteroid [17].

HA plays a role in traumatic energy dissipation and lubrication [18]. IA-HA is capable of decreasing nerve impulses related to OA pain. By benefiting from exogenous HA, endogenous proteoglycan and hyaluronic acid production are improved [19]. HA binds to CD44 on chondrocytes and reduces IL-1β action that decrease activity of MMP-1, 2, 3, 9 and 13 [20]. HA also binds to hyaluronan mediated motility (RHAMM) receptor and could be helpful for chondroprotection [21]. Synovium nitric-oxide production is also inhibited [22]. IA-HA is capable to reduce aggrecan degradation process [23]. IA-HA treatment can inhibit many inflammatory pathways through Toll-Like Receptors reducing TNF-a, IL-1β, IL-6, IL-17, MMP-13 and Nf-kB [24, 25]. IA-HA also affects the sub-chondral bone and its abnormal metabolism [26]. The concentration and molecular weight of IA-HA in OA knee joints are lower than normal [27].

Differences exist in concentration, molecular weight, source of HA (biological fermentation-derived HA or avian-derived HA), dosage (number of injections and intervals), expected duration of effects, cross linkage and added formulations [28]. Based on HA molecular weight, these products are classified in three groups (high ≥3000 kDa, moderate 1500–3000 kDa and low ≤1500 kDa) [29]. Many studies claim that high-molecular-weight intra-articular hyaluronic acids (HMW IA-HA) have better chondro-protective, anti-inflammatory, proteoglycan production, rheologic, analgesic and mechanical properties [30]. They suggest that HMW IA-HA and those biological fermentation-derived HAs probably provide better efficacy and safety [29]. There are a wide-variety of randomized clinical trials (RCTs) and systematic reviews with meta analyses concerning hyaluronic acid efficacy in knee OA [31, 32]; most of which reported beneficial effects in terms of pain and function. In a few studies the efficacy of single cross-linked HMW-HA has been investigated, though there exists a discrepancy between them [33–36]. Our utilized HMW-HA (Arthromac®, Novatex Bioengineering SA Switzerland) is one of these cross-linked products which is indicated for single intra-articular injection in knee OA patients [37].

The aim of current trial was to compare the efficacy and safety of the single cross-linked HMW injection versus triple injection of low-molecular-weight IA-HA among knee OA patients in terms of function and pain improvement during a six-month period.

## Methods

### Participants

In this RCT 90 patients aged between 45 and 75 years suffering from knee OA symptoms lasting for at least 3 months were included. Knee OA was classified according to Kellgren and Laurence score (KLS) [38]. Only subjects with KLS grade of II-III were eligible. The other exclusion criteria were as the followings: breastfeeding or pregnancy, vascular collagen and immunodeficiency disorders, diabetes mellitus, a history of malignancy, body mass index (BMI) > 32 kg/m^2^, mal-alignment as genu varum or valgum greater than 20°, any knee trauma or intra-articular injection during the last 6 months, prior hypersensitivity reaction to avian products or egg protein.

This study protocol was also registered in Iranian database of RCTs (IRCT; www.irct.ir) with the trial registration number IRCT20130523013442N24 and registration date 2018-07-13. Besides, the Ethics Committee of Shahid Beheshti University of Medical Sciences was in charge of approving this study (No: IR.SBMU.MSP.REC.1396.899). A written informed consent was obtained; moreover, a physiatrist described the methodology, probable advantages and disadvantages of HA injections for every participant.

### Interventions

The patients were randomly divided into two groups of 44 and 46 subjects using a computer software for random number generation. In the first group, a HMW-HA (Arthromac®, Novatex Bioengineering SA Switzerland) was administered as a single intra-articular knee injection for 44 participants. HMW-HA solution was provided in a 3 mL prefilled syringe (60 mg of sodium hyaluronate). In the second group, 46 subjects received a low-molecular weight HA (molecular weight 500–730 kDa) (Hyalgan®, Fidia Pharmaceutici S.P.A Italy) as three weekly sessions of IA injection. LMW-HA was provided in a 2 mL prefilled syringe (20 mg of sodium hyaluronate). All injection were performed by an expert physiatrist who had 15 years of experience in intra articular injections. The physician who injected HAs was not blinded. Rather, the assessor physicians who were three senior residents remained unaware to patients’ group till the end.

Our patients did not receive any anti-inflammatory or analgesic agents since 2 weeks before the first injection known as washout period. Prior to injection, routine skin cleansing with the aid of povidone-iodine was performed. Twenty-two gauge (22G) needles through lateral midpatellar approach for knee intra-articular injections were used to administer HMW-HA and LMW-HA in a sterile manner. Upon completion of injections, the participants were requested to flex and extend their knees 10 times. Next, the patients of both groups rested briefly, after which they were given a written protocol of exercises and recommendations to be performed at home. A period of 24–48 h rest along with 20-mintue cold therapy 3 times a day and restricted weight-bearing over knee joints were strongly suggested. The exercise therapy protocol comprised of isometric strengthening workouts that gradually progressed to closed-chain isotonic exercises. Hamstring stretching and muscles strengthening (quadriceps femoris, hip adductor groups, gluteus medius and maximus) were executed three times a day, each time lasting 15 s and repeating 5 times. All patients were followed-up for 8 and 24 weeks after the therapy using visual analog scale (VAS), Lequesne index, and Western Ontario and McMaster Universities Arthritis Index (WOMAC) questionnaire. VAS, WOMAC, and Lequesne indices were employed to investigate the patients’ function and pain at three time-points; once at the baseline and two other times at the 2nd and 6th month after the injections [39–41]. Moreover, minor adverse events such as the injection-induced pain was assessed in both groups. For all indices, lower scores indicate a better condition.

### Statistical analysis

Final data before and after the treatment were imported and analyzed in SPSS v.22. Normality of the data was evaluated using Shapiro–Wilk’s test. Qualitative variables were expressed as frequency and percent. Chi square test was applied to analyze the differences of these qualitative parameters between the two groups. Also, the paired t-test and independent t-test were used to compare mean values within and between the two groups, respectively. In case of non-parametric data, we utilized Friedman and Mann-Whitney U tests, respectively. Statistical significance value was set at *P* < 0.05.

## Results

**Fig. 1:**
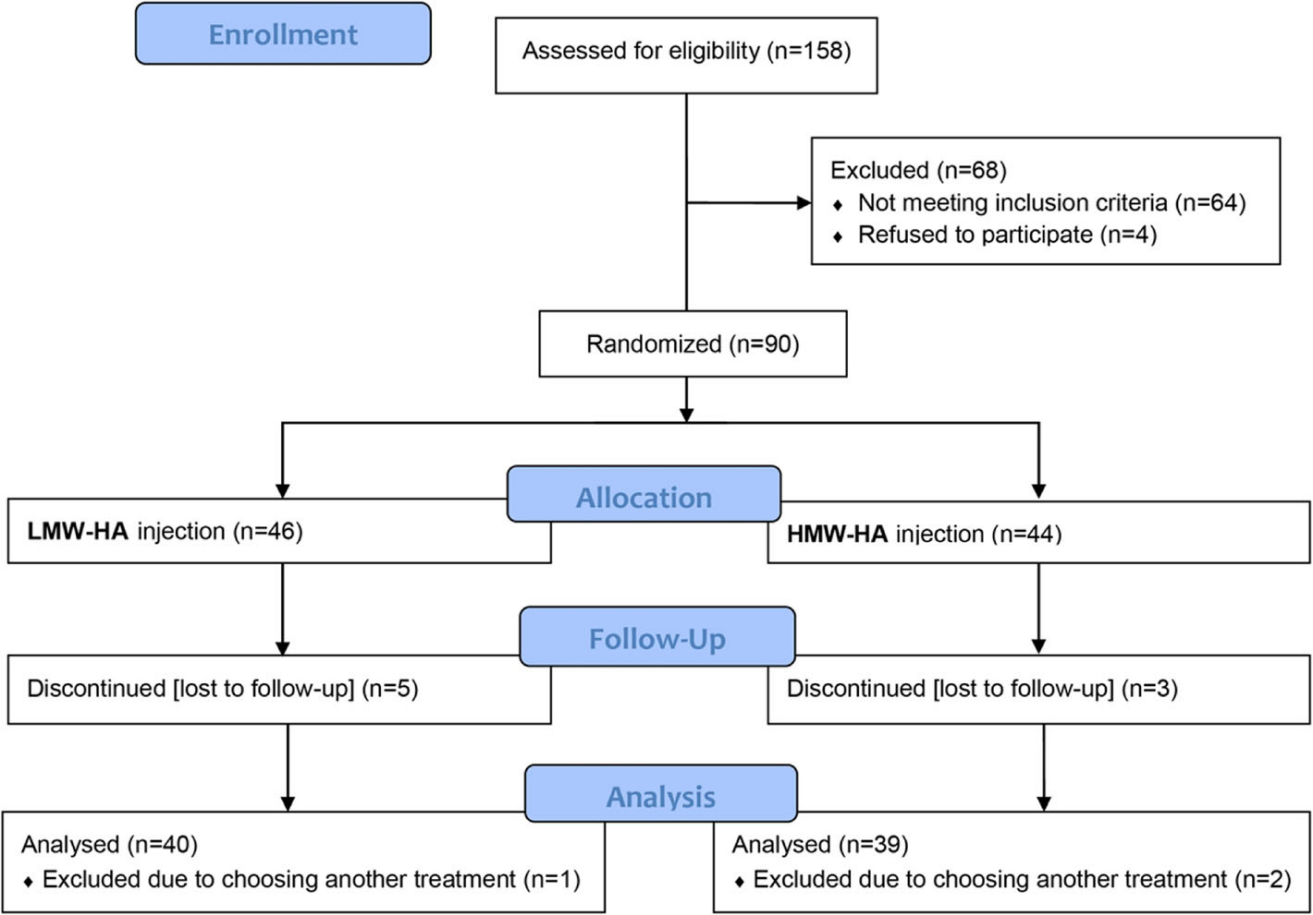
Patient disposition

Among 158 patients with knee OA as candidates for IA-HA injection, 68 patients were excluded from the study. Ninety subjects who met our criteria were randomized to the HMW-HA (44 patients) and LMW-HA (46 patients) groups and received IA-HA in a manner demonstrated in Fig. 1. Eleven participants discontinued the study; therefore, the final number of subjects for the analysis was 39 in the HMW-HA group and 40 in the LMW-HA group. There was no significant difference in baseline characteristics between the two groups (Table 1). The majority of participants were female in both groups (71.8% in HMW-HA and 75% in LMW-HA group).

**Table 1:** Baseline Demographic of the two groups

Variable	HMW-HA* (***N*** = 39)	LMW-HA * (***N*** = 40)	***P*** value
**Age**_**[year] Median (Range)**_	56 (41–66)	59.5 (45–70)	0.305
**Weight**_**[kg] Median (Range)**_	74 (59–98)	75 (57–89)	0.879
**Height**_**Mean (SD)**_	1.66 (0.09)	1.65 (0.07)	0.521
**BMI**_**[kg/m2] Median (Range)**_	27.05 (23–34)	27.45 (22–32)	0.462
**Female**: **Male**_**(%)**_	71.8%: 28.2%	75%: 25%	0.747
**Right**: **Left**_**(Number)**_	21: 18	16: 18	0.21
**KLS Grade ll**: **lll**_**(Number)**_	20: 19	24: 16	0.43

* The values are presented as the median, with the range in parentheses; Abbreviations: *BMI* Body Mass Index, *KLS* Kellgren Lawrence Score**.** ^**#**^ *P* values refer to comparison between the two groups, based on the student’s T-test

Changes in WOMAC, Lequesne index and VAS mean values in each group have been demonstrated in Table 2. Findings showed that all outcome measuring tools statistically improved at 2 months and 6 months of follow-up, compared to the baseline level (*p* < 0.001). there was no significant difference between the LMW-HA and the HMW-HA groups based on three subscales of Lequesne index. Our analysis revealed a similar pattern in VAS mean values. In WOMAC subscale there was no superiority between two groups with one exception in WOMAC stiffness subscale at 2 months follow-up (Table 3). When comparing WOMAC stiffness improvement, LMW-HA was statistically superior to HMW-HA at the 2nd month follow-up (*P* = 0.021). Furthermore, success rates [defined as ≥30% decrease from baseline scores in WOMAC, Lequesne and VAS have been presented in Table 4.

**Table 2:** Comparison of efficacy within the two groups based on changes from the baseline

	WOMAC				Lequesne				VAS
	pain	stiffness	Function	Total	pain	walk	ADL	Total	VAS
**HMW-HA** [Mean]									
Before	9	3	30	42	5	2	5.5	12.5	8
2 months	5	2	19	25	4	1	4.5	10	2
6 months	5	1	17	22	3	1	4	8.5	3
*P value	< 0.001	< 0.001	< 0.001	< 0.001	< 0.001	< 0.001	< 0.001	< 0.001	< 0.001
**LMW-HA** [Mean]									
Before	9	3	30	44	5.5	1	5.5	12.5	8
2 months	5	1	18	25	3	1	4	9	2
6 months	5	1	17	24.5	3.5	1	4.5	9.75	4
*P value	< 0.001	< 0.001	< 0.001	< 0.001	< 0.001	< 0.001	< 0.001	< 0.001^#^	< 0.001

* *P* values refer to changes over time within each group, based on the Friedman test

# *P* values refer to changes over time within each treatment group, based on Repeated Measures

**Table 3:** Comparison of efficacy between the two groups based on their clinical improvement

	HMW-HA	LMW-HA	***P*** value
**Before** [Mean]			
WOMAC	42	44.00	**0.713**^**a**^
Lequesne	12.42	12.50	**0.866**^**b**^
VAS	8	8.00	**0.276**^**a**^
**2 months** [Mean]			
WOMAC	26.03	25.00	**0.59**^**b**^
Lequesne	9.6	8.93	**0.202**^**b**^
VAS	2	2.00	**0.788**^**a**^
**6 months** [Mean]			
WOMAC	24.08	26.55	**0.247**^**b**^
Lequesne	8.97	9.73	**0.126**^**b**^
VAS	3	4.00	**0.411**^**a**^

^a^ *P* values refer to comparison between the two groups, based on the Mann-Whitney test

^b^ *P* values refer to comparison between the two groups, based on the student’s T-test

**Table 4:** Clinical improvement as percent of changes in mean outcome scores over the time-points

	WOMAC (points)				LEQUESNE				VAS
	pain	stiffness	Function	Total	Pain	walk	ADL	Total	VAS
**HMW-HA**									
Before	9.74 (0.22)	2.72 (0.19)	29.92 (1.04)	42.38 (1.24)	5.08 (0.16)	1.67 (0.09)	5.68 (0.11)	12.42 (0.26)	7.82 (0.18)
2 months	5.00 (0.27)	2.65 (0.17)	19.46 (1.12)	26.03 (1.4)	3.95 (0.19)	1.21 (0.09)	4.45 (0.19)	9.60 (0.40)	2.69 (0.22)
6 months	5.05 (0.28)	1.41 (0.19)	17.62 (1.18)	24.08 (1.55)	3.49 (0.22)	1.38 (0.11)	4.10 (0.19)	8.97 (0.44)	3.46 (0.17)
Mean Difference^a^ (SD)	−4.7 (0.32)	−1.3 (0.26)	−12.30 (0.9)	−18.30 (1.23)	−1.6 (0.23)	−0.28 (0.1)	−1.57 (0.2)	−3.45 (0.38)	−4.36 (0.27)
Change^b^ (%) from baseline [SD]	47.77 [2.96]	58.16 [5.56]	41.82 [3.17]	43.76 [3.06]	37.38 [3.63]	29.91 [5.94]	28.29 [3.23]	29.93 [2.77]	55.17 [3.20]
Success Rate^c^ (Number) [%]	31 [79.5]	30 [83.3]	28 [71.8]	25 [64.1]	24 [61.5]	18 [46.2]	13 [33.3]	18 [46.2]	34 [87.2]
*P value	< 0.001	< 0.001	< 0.001	< 0.001	< 0.001	< 0.001	< 0.001	< 0.001	< 0.001
**LMW-HA**									
Before	9.28 (0.26)	2.65 (0.19)	29.88 (1.24)	41.33 (1.65 V)	5.53 (0.15)	1.58 (0.13)	5.63 (0.12)	12.50 (0.36)	8.15 (0.17)
2 months	4.83 (0.23)	1.05 (0.16)	18.40 (1.01)	25.00 (1.33)	3.50 (0.17)	1.15 (0.10)	4.18 (0.15)	8.93 (0.34)	2.65 (0.18)
6 months	5.30 (0.24)	1.00 (0.14)	19.00 (1.05)	26.55 (1.43)	3.75 (0.19)	1.28 (0.11)	4.65 (0.16)	9.93 (0.42)	3.65 (0.18)
Mean Difference^a^ (SD)	−3.97 (0.25)	−1.65 (0.14)	−10.87 (1.07)	−14.77 (1.05)	−1.77 (0.19)	−0.30 (0.11)	−0.975 (0.13)	−2.57 (0.28)	−4.50 (0.25)
Change^b^ (%) from baseline [SD]	42.56 [2.26]	63.20 [4.72]	36.55 [2.52]	35.62 [2.33]	32.62 [2.98]	17.98 [5.12]	19.03 [2.23]	21.21 [2.20]	54.71 [2.67]
Success Rate^c^ (Number) [%]	34 [85]	34 [87.2]	27 [67.5]	29 [72.5]	21 [52.5]	11 [28.9]	7 [17.5]	9 [22.5]	36 [90]
*P value	< 0.001	< 0.001	< 0.001	< 0.001	< 0.001	< 0.001	< 0.001	< 0.001^#^	< 0.001
**P **value**	0.66	0.21	0.94	0.94	0.69	0.53	0.70	0.80	0.48

*P values refer to changes over time within each group, based on the Repeated Measures; **p value between groups, based on the Repeated Measures; ^a^ (6th month-Baseline); ^b^ ([6th month-Baseline]/Baseline) *100; ^c^ for each participant 30% change was considered as the success

Eventually, the frequency of minor complications and injection-induced pain have also been showed in Table 5. Joint stiffness and swelling occurred in 8 (20.5%) patients in the HMW-HA group versus 5 (12.5%) subjects of the LMW-HA group (*P* = 0.378). The mean value of injection-induced pain was 2.64 and 1.9 in HMW-HA and LMW-HA groups, respectively (*P* = 0.286). Fortunately, no systemic adverse event or major complication such as septic arthritis was reported in the present RCT.

**Table 5:** Comparison of adverse-events occurrence between the two groups

	HMW-HA	LMW-HA	***p***-value
**Post injection pain** **Mean (SD)**	2.64 (2.265)	1.90 (1.392)	0.286 ^a^
**Stiffness and heaviness** **Number (Frequency %)**	4 (10.26%)	3 (7.5%)	0.712 ^b^
**Swelling** **Number (Frequency %)**	4 (10.26%)	2 (5%)	0.432 ^b^
**Total** **Number (Frequency %)**	8 (20.51%)	5 (12.5%)	0.378 ^b^

^a^ *P* values refer to comparison between the two groups, based on the student’s T-test

^b^ *P* values refer to comparison between the two groups, based on Chi square test

## Discussion

Based on results of this study clinical improvement with a single cross-linked HMW-HA injection could be relatively equal to that of triple injection of a linear LMW-HA, within the periods of two and 6 months follow-up. Moreover, a comparison between the two groups indicates that there exists no statistically significant superiority. An exception was the improvement of WOMAC stiffness subscale which was significantly higher in LMW-HA group in 2 months.

Altman [29] review which included 68 randomized trials proved that HMW-HA efficacy was superior to LMW-HAs. Conversely in our study, there was no difference in efficacy between these two types of HAs. In the study conducted by Zhang et al. [36], the therapeutic effectiveness of single injection of a cross-linked HMW-HA (Durolane) was compared to five injections of a LMW-HA (Artz), showing that during a period of 26 weeks, Durolane was non-inferior to Artz in terms of pain, physical activity and knee-stiffness. Our study revealed a similar result within a period of same length.

Similarly, Diracoglu et al. [33] evaluated the efficacy of two HA types with different molecular weights and number of injections. The first group received a single cross-linked moderate-molecular-weight HA (Monovisc), while the other one underwent three consecutive weekly injections of a linear LMW-HA (Adant). In both groups, WOMAC scores and VAS-pain showed statistically significant improvements compared to the baseline level, without any remarkable superiority between the two groups. However, in both groups, WOMAC stiffness showed no significant improvement. Meanwhile, VAS improvement for the group receiving Adant was remarkably higher than the Monovisc group. The latter study found that a single cross-linked HA can be as effective as a triple linear LMW-HA, exactly similar to our study. It should be pointed out that the HA used in our trial was much heavier than the one used by Diracoglu. Unlike the mentioned results, WOMAC stiffness in our investigation was associated with a statistically significant improvement. This change was even more evident in the group receiving LMW-HA compared to HMW-HA group.

Another study by Estades-Rubio et al. [35], evaluated a single dose of Durolane versus a five-time GO ON® injection. Mobility and WOMAC were assessed during 6 months. A statistically significant change was observed for both groups compared to their baseline level. In addition, a remarkable superiority was observed in WOMAC scores of the group receiving Durolane compared to the GO ON® group, although no difference was detected in mobility values. From the economic point of view, the total price of using a single injection of Durolane was lower than that of multiple injections of GO ON®. In comparison, the results of our RCT showed some dissimilarities since the improvement in the Durolane as a cross-linked HMW-HA is statistically more significant than GO ON®. However, this finding proves that a single cross-linked HMW-HA can be as effective as or even better than multiple linear LMW-HA injections.

In the meta-analysis conducted by Concoff et al. [34], the efficacy of multiple HA injections versus a single dose of HA was studied. The pooled data showed that a single HA injection was not significantly more effective than IA-Saline in a period of 6 months. Another systematic review and meta-analysis by Zhao et al. [42], was carried out to compare the results of Hylan G-F 20 and LMW-HA in knee OA patients. The final results indicate a similarity between the Hylan G-F 20 and LMW-HA groups in terms of their pain-relief effect. However, Hylan G-F 20 was more effective in pain improvement from 2 to 3 months. It should be pointed out that in the present meta-analysis, Hylan G-F 20 injections were administered more than once; however, this number was less than the number of LMW-HA injections in most trials. These findings, similar to our study, showed the effectiveness of HMW-HA with lower number of injections compared to LMW-HA with multiple injections.

In our study, the rate of minor complications and injection-induced pain was not statistically different between two HA products. In Bannuru’s meta-analysis [43], none of the HA products were significantly different from each other with regard to incidence of adverse events and were relatively equal to IA placebo. Altman et al. [29], concluded that there is no significant difference in the occurrence of effusion across molecular weight subgroups. Different brand names of HA exist in the market, claiming to be effective by a single injection. So far, few studies have been conducted to compare a single HA injection with multiple HA injection. Although most single-injection HAs are of HMW and cross-linked type, some differences can be observed in their structure. To examine the exact effects of such viscosupplements, further well-designed investigations should be performed. Evidently, single injections possess the advantage of lower cost, patients’ comfort and lower risk of complications owing to the lower number of injections.

An advantage of this research is employing various outcome measures for evaluating patients’ symptoms including WOMAC subscales, Lequesne and VAS indices. The washout period was considered in this study. The fact that the physician was not blind in this study, is an important limitation. Due to ethical issues, it was not possible to do second and third sham injections in HMW-HA group. Rather, all assessors in this study were completely blinded. It would be better to enroll a higher number of patients in the future studies. Although our follow-up was for 6 months, yet longer follow-up time can be suggested. As the last limitation to be mentioned, no economic analysis was conducted in our trial.

## Conclusion

Both HMW IA-HA and LMW IA-HA caused significant functional improvement and pain relief; however, there was no significant difference between HMW IA-HA versus three weekly LMW IA-HA in terms of pain relief and function improvement in knee osteoarthritis patients in 6 months of follow-ups. This study revealed that a single HMW-HA injection is as effective as multiple injections of LMW-HA counterparts in periods of 2 and 6 months. Further research into the subject probably sheds lights on choosing the more suited protocol of HA injections.

## Supplementary information

**Additional file 1.** CONSORT 2010 checklist.

## Data Availability

The authors do not intend to share substantial data of this study; but they are ready to share the de-identified file of substantial data in excel format and all other study-related documents, at any specific time for any period, on the demand of editorial board via the corresponding author’s email.

## Abbreviations

HMW-HA: High-molecular-weight hyaluronic acid
LMW-HA: Low-molecular-weight hyaluronic acid
OA: Osteoarthritis
NSAIDs: Nonsteroidal anti-inflammatory drugs
PRGF: Plasma rich in growth factor
PRP: Platelet-rich plasma
IA-HA: Intra-articular hyaluronic acid
RCTs: Randomized clinical trials
MMPs: Matrix metalloproteinases
KLS: Kellgren and Lawrence score
BMI: Body mass index
VAS: Visual analog scale
WOMAC: Western Ontario and McMaster Universities Arthritis Index
